# ETS1 Suppresses Tumorigenesis of Human Breast Cancer via Trans-Activation of Canonical Tumor Suppressor Genes

**DOI:** 10.3389/fonc.2020.00642

**Published:** 2020-05-14

**Authors:** Gi-Cheon Kim, Choong-Gu Lee, Ravi Verma, Dipayan Rudra, Taemook Kim, Keunsoo Kang, Jong Hee Nam, Young Kim, Sin-Hyeog Im, Ho-Keun Kwon

**Affiliations:** ^1^Department of Microbiology and Immunology, Yonsei University College of Medicine, Seoul, South Korea; ^2^Institute for Immunology and Immunological Diseases, Yonsei University College of Medicine, Seoul, South Korea; ^3^Natural Product Informatics Research Center, Korea Institute of Science and Technology (KIST), Gangneung Institute of Natural Products, Gangneung, South Korea; ^4^Academy of Immunology and Microbiology (AIM), Institute for Basic Science (IBS), Pohang, South Korea; ^5^Department of Biological Sciences, Korea Advanced Institute of Science and Technology, Daejeon, South Korea; ^6^Department of Microbiology, College of Natural Sciences, Dankook University, Cheonan, South Korea; ^7^Department of Pathology, Chonnam National University Medical School, Gwangju, South Korea; ^8^Department of Oral Pathology, School of Dentistry, Chonnam National University, Gwangju, South Korea; ^9^Division of Integrative Biosciences and Biotechnology, Department of Life Sciences, Pohang University of Science and Technology, Pohang, South Korea; ^10^Brain Korea 21 PLUS Project for Medical Sciences, Yonsei University College of Medicine, Seoul, South Korea

**Keywords:** *ETS1*, breast cancer, tumor suppressor, DNA methylation, regulatory elements

## Abstract

*ETS1* has shown dichotomous roles as an oncogene and a tumor suppressor gene in diverse cancers, but its functionality in breast cancer tumorigenesis still remains unclear. We utilized the Cancer Genome Atlas (TCGA) database to analyze comprehensive functions of *ETS1* in human breast cancer (BRCA) patients by investigating its expression patterns and methylation status in relation to clinical prognosis. *ETS1* expression was significantly diminished by hyper-methylation of the *ETS1* promoter region in specimens from BRCA patients compared to a healthy control group. Moreover, *ETS1*^high^ BRCA patients showed better prognosis and longer survival compared to *ETS1*^low^ BRCA patients. Consistent with clinical evidence, comparative transcriptome analysis combined with CRISPR/Cas9 or shRNA based perturbation of *ETS1* expression revealed direct as well as indirect mechanisms of *ETS1* that hinder tumorigenesis of BRCA cells. Taken together, our study enlightens a novel function of *ETS1* as a tumor suppressor in breast cancer cells.

## Introduction

Breast cancer is the most commonly diagnosed cancer and the second leading cause of death from cancer in women (1). Development and progress of breast cancer are mediated by a complicated process in which many genes and signaling pathways are intertwined (2). Profiling of gene-expression in patient specimen has been used to identify the key molecular switches of tumor development (3). Human breast cancer datasets, including The Cancer Genome Atlas (TCGA) (4) and Curtis (5) provide comprehensive genomic profiles of breast cancer.

*ETS1* (ETS proto-oncogene 1, transcription factor) has initially been characterized as the proto-oncogenic transcription factor that contributes to tumor angiogenesis and invasiveness in cancer cells (6–8). Previously, high levels of *ETS1* expression have been closely associated with higher chance of metastatic potential and poor prognosis in various types of cancers (9–14). *ETS1* is known to enhance the expression of numerous tumorigenic genes involved in tumor angiogenesis, cancer cell invasion, and energy metabolism (15). These include vascular endothelial growth factor (VEGF) and certain proteases such as MMP-1, MMP-3, and MMP-9, as well as urokinase type plasminogen activator (uPA), which is associated with extracellular matrix (ECM) degradation (16–19). Despite the established oncogenic function of *ETS1* in human cancers, recent studies have proposed contrasting roles of *ETS1* as anti-oncogenes suggesting dichotomous roles of *ETS1* for tumorigenesis in context-dependent manner (20, 21). However, the functionality and molecular action mechanisms of *ETS1* in BRCA tumorigenesis still remain unclear.

In this study, we revealed *ETS1* as the tumor suppressor gene in BRCA cells. In humans, poor prognosis of BRCA patients was negatively correlated with *ETS1* expression, repressed by hyper-CpG methylation in *ETS1* promoter locus. Furthermore, we showed the direct and indirect mechanisms of *ETS1* to hinder tumorigenesis of BRCA cells. Overall, our findings enlighten the novel function of *ETS1* as the tumor suppressor gene, which can be the potential target for novel therapeutics in BRCA.

## Materials and Methods

### Cell Culture, Plasmid, and Reagents

MDA-MB-231 cells were cultured in DMEM (WELGENE: LM 001-05) supplemented with 10% FBS (Gibco: 10099-141) and 100 U/ml of penicillin-streptomycin (Thermo: 15140122). Mutant MDA-MB-231 cells (ΔCRE) harboring deleted promoter region (−540 to −80) of *ETS1* were established using the CRISPR/Cas9 method (22). Mutations were confirmed by Sanger sequencing, and the effect of CRE deletion on *ETS1* level was tested by immunoblotting. Cells were harvested with 0.05% trypsin-EDTA (Gibco: 25300-054). The following chemicals were used; phorbol 12-myristate 13-acetate (PMA, Calbiochem: 524400) and Ionomycin (Calbiochem: 407950).

### Knockdown and Ectopic Expression of *ETS1* by Lentiviral Transduction

Gene knockdown was accomplished using the shRNA system with control shRNA (TR30021) or *ETS1* targeted shRNA (TL313153) (OriGene Technologies, Rockville, MD). MDA-MB-231 cells were exposed to lentiviral concentrates. Gene overexpression was accomplished using Human cDNA clone *ETS1* (RC215203L2) (OriGene Technologies, Rockville, MD). MCF-7 cells were infected with lentiviral particle encoding h*ETS1*. After lentiviral transduction, cells were harvested and sorted with Moflo XDP (Beckman Coulter, Fullerton, CA) for GFP negative and positive cells.

### RNA Isolation, cDNA Synthesis, and Quantitative RT-PCR

Total RNA was extracted using a TRI Reagent (Molecular Research Center) following standard protocols. Reverse transcription of 1 μg RNA was performed using oligo (dT) primer (Promega: C1101) with Improm II Reverse Transcription system (Promega) according to the manufacturer's protocols. Quantitative RT-PCR was performed using SYBR Green Dye mix (Takara: RR420) on Rotor-Gene Q (Qiagen, Hilden, Germany). Data was normalized to human hypoxanthine-guanine phosphoribosyl transferase (*HPRT*). Primer sequences are provided in Supplementary Table 1.

### Chromatin Immunoprecipitation (ChIP)-PCR Assay

ChIP-PCR assays were performed by Simple ChIP plus Enzymatic Chromatin IP Kit (Cell Signaling: #9005) according to the manufacturer's protocols. Briefly, cells were cross-linked with 1% of formaldehyde and lysed for nuclei preparation. Nucleus pellet was treated with micrococcal nuclease and sonicated for chromatin fragmentation. Protein-chromatin complex was incubated with antibody targeting anti-*ETS1* (Cell Signaling: #14069) at 4°C overnight. Rabbit IgG (Vector Laboratories) was used as negative control. After immuno-precipitation, 50 μ Dynabeads protein G or A (Life technologies) were added and rotated further for 6-h at 4°C. Ab/protein/chromatin complex were reverse-crosslinked at 65°C overnight, and DNA was purified by DNA purification columns (Cell Signaling: #10010). The relative enrichment of specific regions in precipitated DNA was measured by quantitative PCR (qRT-PCR). To quantify protein binding in specific genomic locus, purified DNA was used for qRT-PCR. Primer sequences are listed in Supplementary Table 2.

### Immunoblot Assay

Whole cell lysates were extracted using RIPA buffer according to manufacturer's protocols. Protein concentration was measured by Bradford protein assay (Bio-Rad: #5000001), and 20 or 30 μg of proteins were used for SDS-PAGE (10%) and then transferred onto a nitrocellulose membrane (Bio-Rad: 162-0097). The following primary antibodies targeting ETS1 (Santa Cruz Biotechnology: sc-55581) and ACTIN (Abcam: ab3280) were used. Protein expression was visualized with ImageQuant™ LAS 4000 (GE healthcare Life Science, Piscataway, NJ). ACTIN expression was used as a loading control for whole cell lysates.

### Flow Cytometric Analysis

MDA-MB-231 (WT) and ΔCRE cells were harvested, washed with PBS, fixed by 2 ml of cold 70% ethanol dropwise, and incubated at −20°C overnight. For checking proliferation by Ki-67, diluted anti-Ki-67 antibody (BioLegend: #652404) was added and incubated at room temperature (RT) for 30 min in the dark. After incubation, cells were washed and re-suspended in 200 μl of PBS. Cells were then analyzed with BD LSRFortessa (BD Biosciences, San Jose, CA) and FlowJo software (Treestar, San Carlos, CA).

### Xenograft Cancer Model

Six-week-old female nude mice (Orient Bio) were injected subcutaneously with MDA-MB-231 (5 × 10^6^) or ΔCRE cells (5 × 10^6^) to the left or right abdomen, respectively. In addition, nude mice were injected subcutaneously with MCF-7 GFP negative (*ETS1*^−^) or MCF-7 GFP positive (*ETS1*^+^) (5 × 10^6^) to the left or right abdomen, respectively. Tumor size was measured every 4 days by caliper measurements. All mice were housed in a specific pathogen-free barrier facility, and allocated and randomized for the experiments by a technician at the animal facility. The experiments were performed in accordance with protocols approved by POSTECH Institutional Animal Care and Use Committee, Korea. All experiments were performed in accordance with relevant guidelines and regulations.

### RNA-Sequencing

Total RNA was extracted and purified with RibospinTMII (GeneAll biotechnology: 314-150). RNA was subjected to library preparation with TruSeq Stranded mRNA Sample Preparation Kit (Illumina: RS-122-2101~2), and RNA-sequencing was performed by NextSeq 500 Sequencing System (Illumina, San Diego, CA). Sequences were mapped to hg19 with TopHat (version 2.0.12). Estimated expression level was generated with Cufflinks (version 2.2.1), and differentially expressed genes were selected using Cuffdiff (version 2.2.1). RNA-seq was performed on two biological replicates. The set of differential expressed genes were analyzed using DAVID gene functional classification tool (23).

### Dataset for Human Samples and Human Cell Lines

TCGA (The Cancer Genome Atlas) data were analyzed using a web-based program. Wanderer Website (http://gattaca.imppc.org:3838/wanderer/) was used to determine differential expressions of *ETS1*, target genes, and methylation profiles in the tumor and normal specimens (24). OncoLnc (http://www.oncolnc.org/) was used to calculate survival rate according to *ETS1* expression level in tumor samples (25). Top or bottom 10% of total patients in ETS1 expression or methylation level was selected for further analysis. The cBioPortal (http://www.cbioportal.org/) was used to determine the correlation between *ETS1* alteration and survival rate in tumor samples.

### Statistical Analysis

Error bars indicate standard deviation (SD). All *t*-tests performed were student two-tailed tests (^*^P < 0.05, ^**^P < 0.01, and ^***^P < 0.001).

### Data Accessibility

RNA-seq datasets have been deposited in GEO database with accession code GSE106634.

## Results

### Clinical Relevance of *ETS1* Expression in Breast Cancer Patients

**Figure 1:**
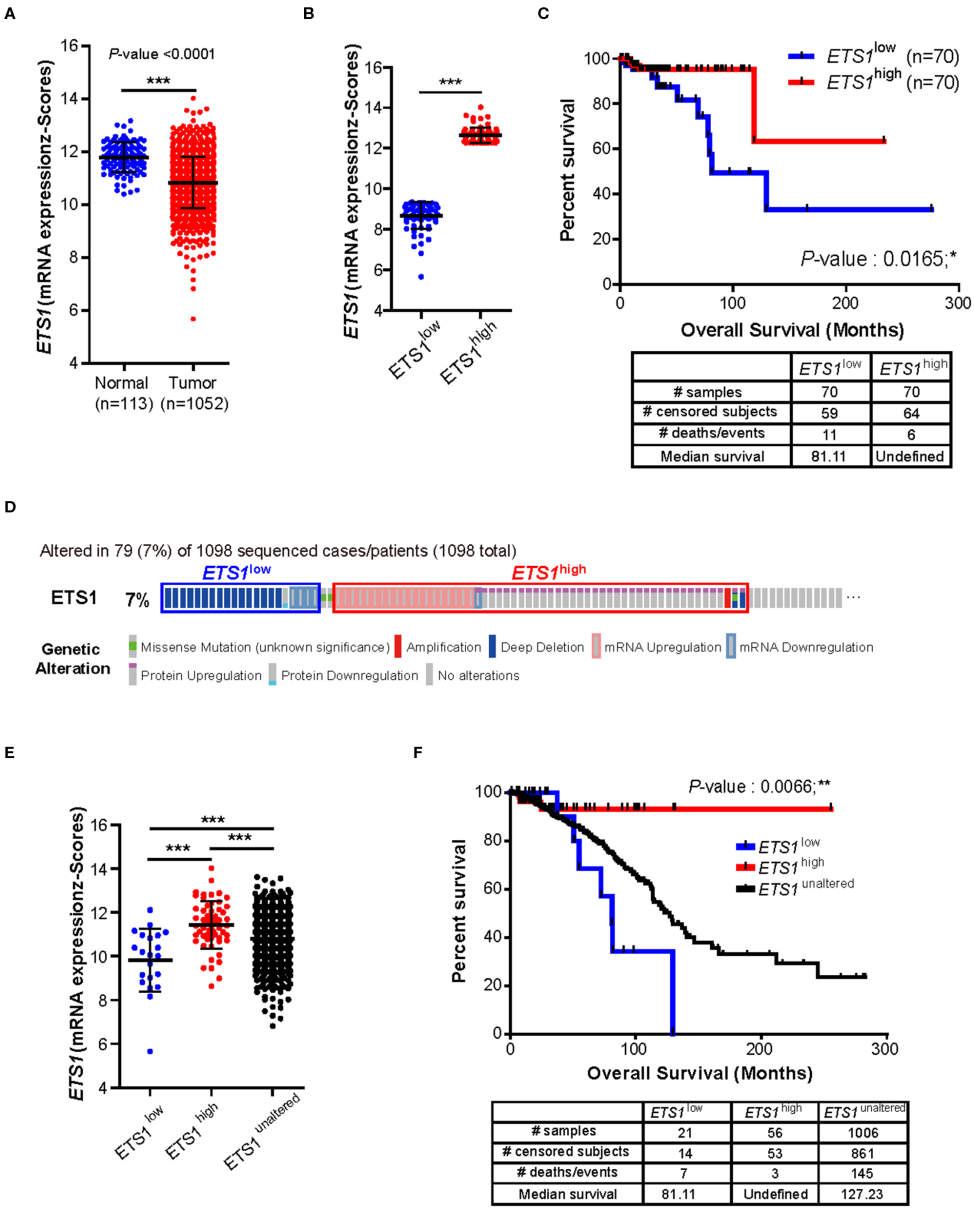
Clinical implication of *ETS1* level in breast cancer patients. **(A)** Comparison of *ETS1* expression between normal (*n* = 113) and BRCA patient (*n* = 1052) specimens in publicly available datasets (TCGA). Each symbol represents an individual; horizontal lines indicate the mean. ***P < 0.001 (Student *t*-test). **(B)** *ETS1* expressions of *ETS1*^low^ (*n* = 70) and *ETS1*^high^ (*n* = 70) groups. **(C)** Correlation between *ETS1* expression and survival rate. A total of 1,052 samples were divided into *ETS1*^high^ (*n* = 70) and *ETS1*^low^ (*n* = 70) groups according to *ETS1* expression levels. Overall survival analysis was performed based on *ETS1* levels in BRCA patients. Blue and red lines indicate patients with *ETS1*^low^ (*n* = 70) and *ETS1*^high^ expression (*n* = 70), respectively. *P*-value was calculated using log-rank test (*P* = 0.0165). **(D)** Profiles of genetic alterations of *ETS1* in the 1,098 human BRCA specimens in TCGA dataset. **(E)** *ETS1* expression levels of three groups (unaltered, group 1, and group 2). **(F)** Overall survival rates in three groups (unaltered, group 1, and group 2) of breast cancer patients. Black line indicates unaltered group. Blue and red lines indicate patients with *ETS1*^low^ (*P* = 0.0551) and *ETS1*^high^ expression (*P* = 0.0452), respectively, compared to unaltered group. *P*-values were calculated by log-rank test. *P < 0.05, **P < 0.005.

To determine whether *ETS1* acts as a pro- or anti-tumorigenic factor in BRCA tumorigenesis, we performed correlative analysis of *ETS1* expression with clinical outcome of BRCA patients. Through the analysis of The Cancer Genome Atlas (TCGA) database, we found that, in contrast to previous studies (10, 13, 26–29), breast cancer specimens (*n* = 1,052) significantly reduced *ETS1* expression compared to normal tissues (*n* = 113, Figure 1A). This observation was further validated by analyzing another large clinical dataset, Curtis, that contained 2,000 breast tumor samples (5). Consistently, significantly curtailed expression of *ETS1* in breast cancer specimens (*n* = 1,992) was observed in BRCA specimens compared to normal specimens (*n* = 144; Supplementary Figure 1A). Next, to assess whether *ETS1* expression level has clinical implications, we classified breast cancer patients into two groups based on *ETS1* expression in BRCA specimens (*ETS1*^high^ and *ETS1*^low^; Figure 1B). Intriguingly, we found that BRCA patients in the *ETS1*^*l*^^ow^ group were significantly more likely to have a poor prognosis in TCGA (*P* = 0.0165) as well as the Curtis (*P* = 0.0076) database (Figure 1C and Supplementary Figures 1A,B), compared to BRCA patients in *ETS1*^high^ group not in triple-negative type specific manners (Supplementary Figures 1C,D) suggesting the potential of *ETS1* as the anti-tumorigenic factor in breast cancer. To confirm whether *ETS1* has anti-oncogenic function in general or in specific types of tumor, we extended previous analysis to other cancers as well. Intriguingly, significantly reduced expression of *ETS1* was observed in not all but specific types of cancers, including Bladder Urothelial Carcinoma (BLCA), colon adenocarcinoma (COAD), and Lung adenocarcinoma (LUAD), compared to normal specimens (Supplementary Figure 2A), which is highly correlated with poor clinical outcomes in general, consistent with BRCA (Supplementary Figure 2B). Collectively, these results suggest anti-tumorigenic functions of *ETS1* in a tumor-dependent manner. Next, we questioned whether low levels of *ETS1* in BRCA patients is associated with genetic alterations around *ETS1* genomic locus compared to healthy counterparts, using cBioPortal (Computational Biology Center, Memorial Sloan Kettering Cancer Center). We found that about 7% of BRCA patients (79 among 1,098) in TCGA dataset showed genetic alterations in the *ETS1* gene including amplification or deep deletion, which led to down- or up-regulation of *ETS1* levels, respectively (Figure 1D). Hence, we then divided the patients into three groups, based the effect of generic alteration of *ETS1* on its expression; *ETS1*^low^, *ETS1*^high^, and *ETS1*^unaltered^ (Figure 1E), and assessed the effect of genetic alteration on clinical outcomes in BRCA patients. Compared to *ETS1*^unaltered^, genetic alterations triggering diminished *ETS1* expression (*ETS1*^low^) were highly correlated with poor clinical outcomes, while *ETS1*^high^ showed increased survival rate (Figure 1F). Altogether, these clinical results indicate *ETS1* as a novel tumor suppressor gene in BRCA.

### The Effects of DNA Methylation Status on *ETS1* Promoter Region for Its Expression in BRCA Patients

**Figure 2:**
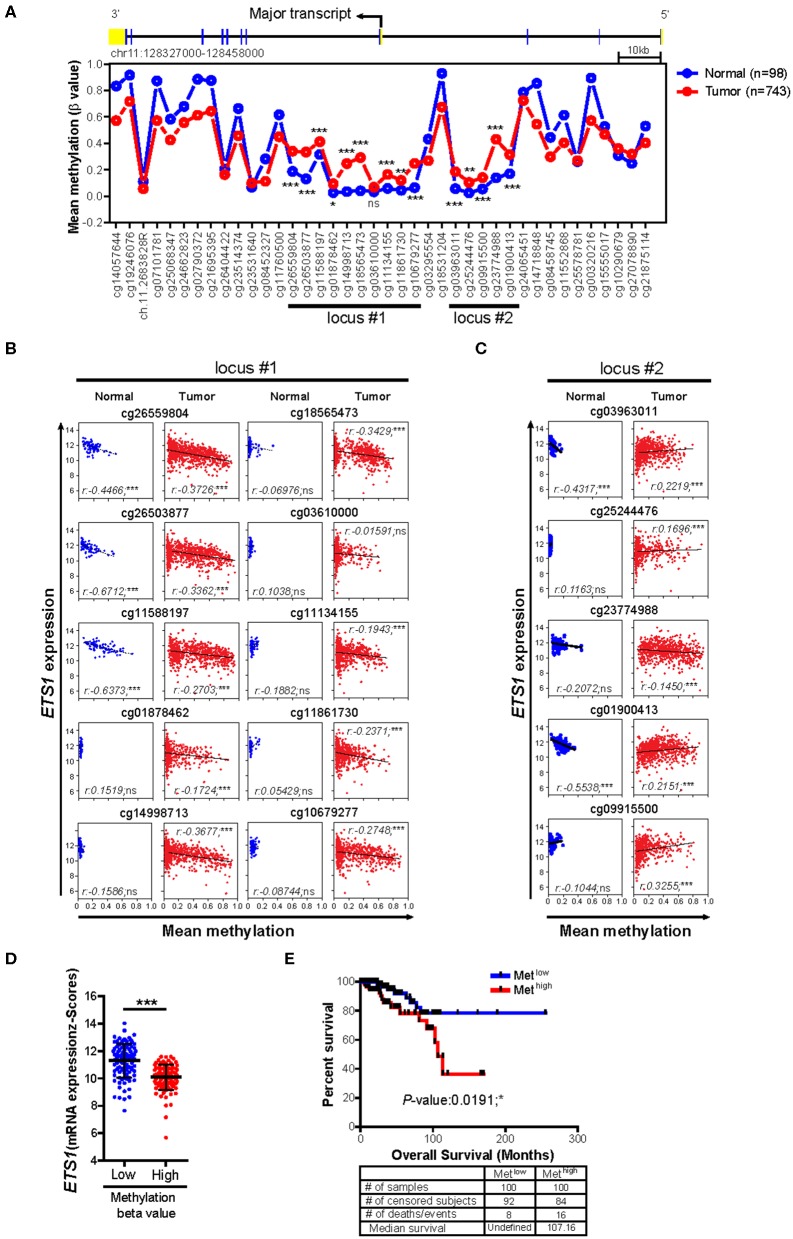
Impact of DNA methylations status of *ETS1* promoter on its expression in BRCA patients. **(A)** Mean methylation (β value) on CpG sites of *ETS1* locus between normal (*n* = 98) and BRCA patient (*n* = 743) specimens by TCGA Wanderer tool. Blue and red lines indicates normal or BRCA patients, respectively. *P < 0.05, **P < 0.01, ***P < 0.001 (two-way ANOVA with Bonferroni test). **(B,C)** Scatterplots depicting linear regression (black line) and Pearson correlation analysis with corresponding *P*-values. Correlation between *ETS1* expression and methylation status represented by β value on each CpG site of locus #1 **(B)** and locus #2 **(C)** in normal and tumor samples. Each symbol represents an individual human specimen. **(D)** *ETS1* expression levels between Met^low^ vs. Met^high^ groups in cg26559804. Blue or red dot indicates methylation low (Met^low^; *n* = 100) or methylation high (Met^high^; *n* = 100), respectively. **(E)** Overall survival rates in two groups (Met^low^ vs. Met^high^) of breast cancer patients. Blue and red lines indicate patients with Met^low^ and Met^high^ (*P* = 0.0191), respectively. *P*-values were calculated using log-rank test.

Next, we questioned how breast tumor cells maintain lower *ETS1* expression than normal cells. To answer this, we decided to focus on DNA methylation, which is one of the major epigenetic mechanisms to control gene expression (30, 31), by comparing methylation status of CpG sites on *ETS1* genomic locus between BRCA patients (*n* = 732) and healthy counterparts (*n* = 84) (24). Intriguingly, we found that tumor specimens showed significantly higher methylation levels in two CpG island regions, located around the promoter of *ETS1* (locus #1 and locus #2), compared to normal specimens (Figure 2A and Supplementary Table 3). Then, we determined whether methylation status of *ETS1* promoter regions is accompanied with *ETS1* expression level. Hyper-methylation in locus #1 but not #2 of *ETS1* promoter region was closely associated with decreased *ETS1* expression in BRCA specimens, indicating inverse correlation of *ETS1* methylation status with its expression (Figures 2B,C). Among 10 CpG sites in locus #1, methylation status of cg26559804, cg26503877, and cg11588197 showed significant inverse correlation with *ETS1* expression in multiple types of cancers (Supplementary Figure 3). Moreover, seven remaining CpG methylation sites seemed to be BRCA-specific, since other tumors showed comparable CpG methylation patterns in normal and tumor specimens (Supplementary Figure 3). Next, we examined the methylation level of the *ETS1* promoter (Met^low^ vs. Met^high^) and its clinical outcomes in BRCA patients. Consistent with *ETS1* expression, enhanced CpG methylation (Met^high^) was correlated with low *ETS1* level (Figure 2D), resulting in poor prognosis (Figure 2E). Collectively, these results suggest that DNA methylation on *ETS1* promoter locus is a key factor to determine *ETS1* expression and disease prognosis in BRCA patients.

### *ETS1* Regulates Growth and Proliferation of Tumor Cells

**Figure 3:**
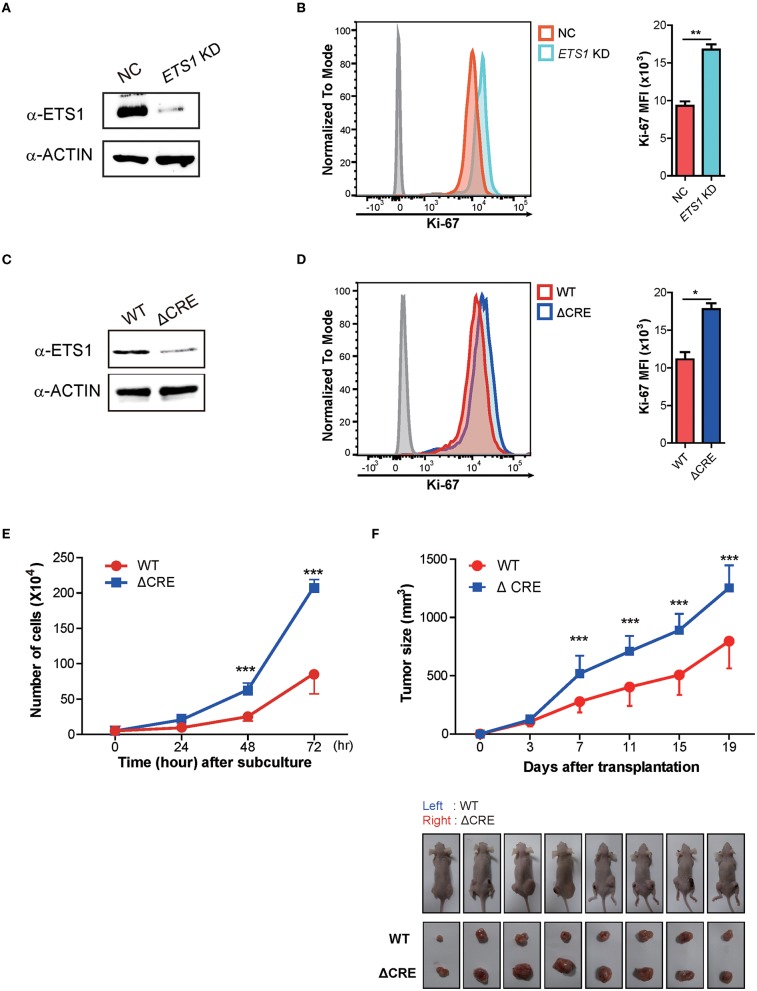
ETS1 inhibits the proliferation and growth of breast cancer cells *in vitro* as well as *in vivo*. MDA-MB-231 cells were transfected with negative control (NC) and *ETS1* shRNA-GFP reporter vector, and transfected cells were sorted according to GFP-expression. The FACs plots are representative of data from three independent experiments. **(A)** Immunoblotting analysis for *ETS1* protein levels in NC and knockdown cells. **(B)** Analysis of cell proliferation (Ki-67) comparing NC and *ETS1* KD cells. **(C)** Immunoblotting analysis for levels of *ETS1* in MDA-MB-231 (WT) cells and ΔCRE cells. **(D)** Quantification of cell proliferation based on detection of Ki-67 protein by flow cytometry. **(E)** Cell count for 72-h culture periods. *P < 0.05, **P < 0.01, ***P < 0.001 (two-way ANOVA with Bonferroni test). **(F)** Tumor growth curves in nude mice after injection of WT (left) and ΔCRE (right) cells. Tumor volumes were measured at an interval of 4 days (*n* = 10). Images of representative tumor-bearing mice (top) and isolated tumors (bottom). *P < 0.05, **P < 0.01, ***P < 0.001 (two-way ANOVA with Bonferroni test). Data is representative of three independent experiments. For reasons of clarity, blots were cropped to the bands of interest. Samples derived from the same experiment and gels/blots were processed in parallel. See full-length blots in Supplementary Figure 6.

The abovementioned findings on BRCA patients support *ETS1* as a tumor suppressor in breast cancer. To elucidate how *ETS1* affects tumorigenesis of BRCA, we tested the effects of knockdown of *ETS1* on tumorigenicity of breast cancer cells. To achieve this goal, we employed MDA-MB-231 cells, well-characterized breast cancer cell lines with high levels of *ETS1* expression (32). MDA-MB-231 cells were transfected with mock and *ETS1* targeting shRNA vector tagged with GFP and found around 70% diminished ETS1 protein level in ETS1-shRNA^+^ cells compared with mock-shRNA^+^ cells (Figure 3A). Interestingly, knockdown of *ETS1* expression significantly increased proliferation capacity of breast cancer cells measured by Ki-67 expression (Figure 3B). To confirm previous observations, we generated mutant MDA-MB-231 cells, in which a core regulatory element (CRE; −540 to −80) of *ETS1* promoter was deleted by CRISPR/Cas9 methods, and named as “ΔCRE” (22). Similar to shRNA based knockdown system, ΔCRE cells demonstrated decreased expression of *ETS1* with enhanced proliferation and growth rate (Figures 3C–E) suggesting anti-tumorigenic function of *ETS1* in BRCA cells. To further validate anti-tumorigenic roles of *ETS1 in vivo*, WT or ΔCRE cells were subcutaneously transplanted into the left or right sides of flank using nude mice and monitored tumor growth *in vivo*, respectively. Indeed, mice engrafted with ΔCRE cells developed significantly larger volume of tumor compared to mice injected with WT cells (Figure 3F). Since MDA-MB-231 is a triple negative breast cell line that can only represent a specific subset of breast cancer, we tried to confirm whether ETS1 inhibits the proliferation of tumor cells with MCF-7 cells (ER^+^, PR^+^ and HER2^−^ cell; Supplementary Figure 4). Ectopic expression of ETS1 in MCF-7 cells significantly reduced cell proliferation *in vitro* (Supplementary Figure 4A) and suppressed tumor growth *in vivo* (Supplementary Figure 4B). Altogether, these results indicate that *ETS1* suppresses proliferation and growth of breast cancer cells *in vitro* as well as *in vivo*.

### *ETS1* Regulates Expression of Cell Proliferation-Related Factors

**Figure 4:**
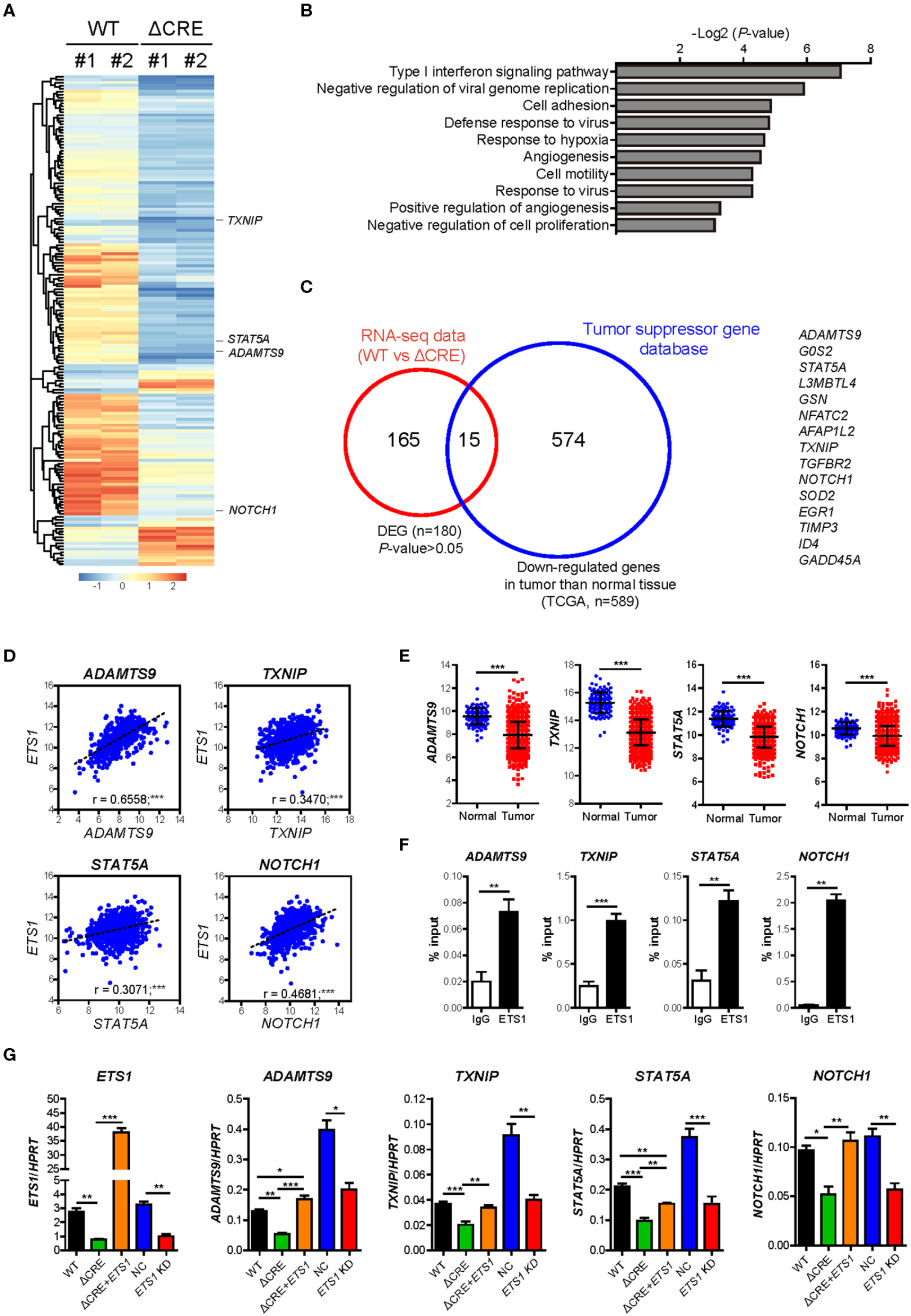
*ETS1* resultes muti-turmogeneic pathways in breast cancer cells. Transcriptome datasets from WT and ΔCRE cells were visualized by **(A)** heatmap analysis and further applied in **(B)** biological process of Gene Ontology (GO) enrichment analysis. **(C)** Venn diagram depicting the overlap of differentially expressed genes (DEGs) together with down-regulated genes in tumor compared to normal tissue, based on TSGene (33), a web resource for tumor suppressor genes. **(D)** Scatterplots depicting linear regression (black dot line) and Pearson correlation analysis with corresponding *P*-values. Correlation between *ETS1* and target genes in BRCA. Each symbol represents an individual human specimen. **(E)** mRNA expression of *ETS1* target genes in normal and tumor specimens. **(F)** ChIP assay was performed using anti-*ETS1* antibody with MDA-MB-231 cells. Relative enrichments were determined by qRT-PCR with primers specific for *ETS1* binding sites in the target gene locus. Representative data from three independent experiments. **(G)** Validation of representative target genes by qRT-PCR. Representative data from three independent experiments. *P < 0.05, **P < 0.01, ***P < 0.001 (One-way ANOVA with Bonferroni test). *P < 0.05, **P < 0.01, ***P < 0.001 (Student *t*-test).

To determine how *ETS1* regulates proliferation and growth of breast cancer cells, we performed an RNA-sequencing (RNA-seq) based transcriptome analysis with WT and ΔCRE cells (Figure 4A and Supplementary Table 4). Among 180 differentially expressed genes (DEG), 80% of DEGs were down-regulated in ΔCRE cells compared to WT cells, indicating a major role of *ETS1* in BRCA as transcriptional activator. To enlighten down-stream pathways affected by *ETS1* in breast cancer cells, we performed Gene Ontology (GO) and found that various tumorigenic pathways, including cell adhesion, angiogenesis, and proliferation, were significantly altered in ΔCRE cells (Figure 4B and Supplementary Table 5). Intriguingly, we noticed that several well-known tumor suppressor genes were significantly under-expressed in ΔCRE cells, of which expression levels were also reduced in BRCA tumor tissue compared to normal tissue from TCGA (Figures 4A,C and Supplementary Tables 6, 7) (33). Furthermore, we found a strong correlative expression between *ETS1* and these tumor suppressor genes (*ADAMTS9, TXNIP, STAT5A*, and *NOTCH1*) in BRCA specimen samples that were actually under-expressed in BRCA compared to normal tissue (Figures 4D,E), suggesting the potential mechanism of *ETS1* as the tumor suppressor through direct activation of other tumor suppressor genes in BRCA cells. Indeed, we found highly conserved putative *ETS1* binding sites on the promoter regions of *ADAMTS9, TXNIP, STAT5A*, and *NOTCH1* using ECR browser (Supplementary Figure 5), and the direct binding of *ETS1* in these genomic loci was confirmed by ChIP assay (Figure 4F) in BRCA cells. To elucidate the effects of *ETS1* binding on these loci, we first knockdowned *ETS1* expression and checked its impact on transcriptional change of these tumor suppressor genes in MDA-MB-231 cells. Consistent with ΔCRE cells, knockdown of *ETS1* significantly attenuated the expression level of these tumor suppressor genes (Figure 4G). Furthermore, knockdown effects of *ETS1* were confirmed by rescuing *ETS1* expression in ΔCRE cells that significantly enhanced the expression of ADAMTS9, TXNIP, STAT5A, and NOTCH1 compared to ΔCRE cells (Figure 4G). Altogether, these data indicate dichotomous functions of *ETS1* as anti-tumorigenic factors through direct activation of targets but also trans-activating other tumor suppressor genes in breast cancer cells.

## Discussion

In this study, we defined *ETS1* as the tumor suppressor in BRCA together with detailed molecular action mechanisms. Analysis of human breast cancer specimens showed a lower level of *ETS1* through hyper-DNA methylation on the *ETS1* promoter region in BRCA compared to normal specimens, which was closely correlated with poor prognosis in a large number of BRCA patients. Furthermore, we elucidated the action mechanisms of *ETS1* as a tumor suppressor not only by directly activating down-stream targets linked with tumor cell proliferation/growth but also trans-activation of other tumor suppressor genes, such as *ADAMTS9, TXNIP, STAT5A*, and *NOTCH1*.

Previous studies have shown dual functions of *ETS1* as both pro-oncogenic (9–13) and anti-oncogenic (19, 20, 31) factors. How does *ETS1* have paradoxical roles in tumorigeneisis? Interestingly, we found a distinct expression pattern of *ETS1* depending on tumor types. For example, unlike BRCA, high level of *ETS1* was observed in cancer specimens from GBM, HNSC, KIRC, PCPG, SARC, and THCA; while other tumor types, including BRCA, showed low levels of *ETS1* compared to normal specimens (Supplementary Figure 2A, data not shown). In addition, we have shown that *ETS1* directly activates several invasiveness factors, such as ENG and MMP14 (Supplementary Table 5) enhancing invasive phenotypes *in vitro* as well as *in vivo* (22). Similarly to *ETS1*, various factors are thought to have dual roles, as tumor suppressor and activator, in a cell type-dependent manner; however, how they switch between the two functionalities has never been established. For instance, PGC-1α, a master regulator of energy metabolism, has recently been shown to exert anti-metastatic effects in cancer through inhibition of EMT, but it also plays the opposite role in specific cancer subtypes by providing growth advantages (34). In addition, TGFβ has shown its anti-tumorigenic function at early stage of cancer, while it supported tumor metastasis in later stages of cancer (35). Furthermore, it is intriguing that ΔCRE cells decreased the expression of gene sets involved in immune responses, such as Type I interferon signaling (*OAS-1, -3* and *IFIT-1, -2, -3*, etc.) and anti-viral responses (*TREM183, BNIP3* etc.) which suggest potential roles of *ETS1* to modulating immunogenicity of BRCA cells. Hence, it is essential to understand how *ETS1* has dichotomous roles in tumorigenesis, which is currently under investigation.

Our comparative transcriptome analysis between MDA-MB-231 cells (WT) and ΔCRE cells has clearly revealed that *ETS1* is directly involved in multiple steps to hinder tumorigenesis including angiogenesis, cell survival, proliferation, and even cell adhesion (Figure 4B). To take a step forward from this analysis, we have identified a unique feature of *ETS1* to inhibit tumorigenesis of BRCA cells by directly trans-activating core regulators of tumorigenesis as tumor suppressor genes in BRCA cells. ADAMTS9 (36, 37) and TXNIP (38) have been shown to inhibit cancer cell proliferation, and STAT5A and NOTCH1 are well-known for their versatile roles to suppress proliferation, survival, differentiation, or senescence of cancer cells (39–42). *ETS1* directly bound in genomic loci of these regulators (Figure 4F) and activated their transcription in breast cancer cells (Figure 4G). Hence, our results enlightened that *ETS1* exerts its anti-tumorigenic function in at least two distinct ways: (i) direct activation of gene-sets involved in BRCA tumorigenesis, and (ii) indirect inhibition of tumorigenesis through trans-activation of canonical tumor suppressor genes in BRCA cells.

Previously, transcription factors (TFs) were implicated in a majority of human diseases, such as cancer and autoimmune diseases. In addition, TFs were considered to be “undruggable” targets, except for ligand-inducible nuclear receptors (43). However, numerous recent cases have successfully targeted TFs, suggesting TFs to be feasible targets for drug development (43). Since *ETS1* level is strikingly correlated with a patient's prognostic status, *ETS1*-targeted therapy seems attractive. Previously, our group and others have identified up-stream signaling pathways and major transcriptional activators for *ETS1* transcription in breast cancer cells (22, 44). In addition, previous studies have shown the importance of post-translational modification (phosphorylation, acetylation, sumoylation, and ubiquitination) and co-factor interaction (45). In this context, our result proposed potential therapeutic approach targeting cis-regulatory elements of *ETS1* to modulate the overall level in cancer cells. Therefore, in the future, we will direct our studies to investigate these mechanisms for the development of therapeutics targeting *ETS1* in BRCA.

In this study, we defined *ETS1* as a tumor suppressor that inhibits growth and proliferation of breast cancer cells in both humans and mice. Our study indicated that *ETS1* might be a good therapeutic target for BRCA due to its intrinsic and extrinsic propensities to inhibit tumorigenesis. Moreover, given the intense interest in understanding the biomarkers for predicting breast cancer prognosis, our findings indicate that evaluating *ETS1* level in tumors may be an important predicator for BRCA patients.

## Data Availability Statement

RNA-seq datasets have been deposited in the NCBI Gene Expression Omnibus with accession code GSE106634.

## Ethics Statement

This study was carried out in accordance with the principles of the Basel Declaration and recommendations of International Association of Veterinary Editors guidelines, Pohang University of Science and Technology (POSTECH) Institutional Animal Care and Use Committee. The protocol was approved by POSTECH Institutional Animal Care and Use Committee.

## Author Contributions

S-HI, G-CK, and H-KK designed the study and wrote the paper. G-CK, C-GL and RV performed the experiments. TK and KK analyzed RNA sequencing data. YK and JN provided intellectual contributions. S-HI, DR, C-GL, and H-KK edited the paper.

## Conflict of Interest

The authors declare that the research was conducted in the absence of any commercial or financial relationships that could be construed as a potential conflict of interest.
