# Supplementary material for: ETS1 Suppresses Tumorigenesis of Human Breast Cancer via Trans-Activation of Canonical Tumor Suppressor Genes
Source: Front Oncol. 2020 May 14;10:642. doi: 10.3389/fonc.2020.00642 (PMC7239993; doi:10.3389/fonc.2020.00642)
Supplement: Supplementary file 8 [file Data_Sheet_1.pdf]

ETS1 suppresses Tumorigenesis of Human Breast Cancer via Trans-Activation of Canonical Tumor Suppressor Genes

Gi-Cheon Kim[1,2], *Choong-Gu Lee*[3], *Ravi Verma*[4], *Dipayan Rudra*[4], *Taemook Kim*[5], *Keunsoo Kang*[6], *Jong Hee Nam*[7], *Young Kim*[8], *Sin-Hyeog Im*[4,9] and *Ho-Keun Kwon*[1,2,10]*

[1] *Department of Microbiology and Immunology, Yonsei University College of Medicine, Seoul, South Korea,* [2] *Institute for Immunology and Immunological Diseases, Yonsei University College of Medicine, Seoul, South Korea*, [3] *Natural Product Informatics Research Center, Korea Institute of Science and Technology (KIST), Gangneung Institute of Natural Products, Gangneung, South Korea*, [4] *Academy of Immunology and Microbiology (AIM), Institute for Basic Science (IBS), Pohang, 37673, Republic of Korea*, [5] *Department of Biological Sciences, Korea Advanced Institute of Science and Technology, Daejeon, South Korea*, [6] *Department of Microbiology, College of Natural Sciences, Dankook University, Cheonan, South Korea*, [7] *Chonnam National University Medical School, Gwangju, South Korea*, [8] *Department of Oral Pathology, School of Dentistry, Chonnam National University, Gwangju, South Korea*, [9] *Division of Integrative Biosciences and Biotechnology, Department of Life Sciences, Pohang University of Science and Technology, Pohang, South Korea*, [10] *Brain Korea 21 PLUS Project for Medical Sciences, Yonsei University College of Medicine, Seoul, South Korea*

*Correspondence: HK@yuhs.ac

***Address correspondence to:** Dr. Ho-Keun Kwon, Department of Microbiology and Immunology, Yonsei University College of Medicine; Brain Korea 21 PLUS Project for Medical Sciences, Institute for Immunology and Immunological Diseases. 50-1 Yonse-ro, Seodaemin-gu, Seoul, South Korea, 03722; Tel: 82-2-2228-1818; Email: HK@yuhs.ac

Supplementary Information

Supplementary Figure 1. Comparison of *ETS1* expression between normal and tumor patient specimens in publically available dataset (Curtis).

Supplementary Figure 2. Comparison of *ETS1* expression between normal and tumor patient specimens in diverse cancer types.

Supplementary Figure 3. DNA methylation status on *ETS1* promoter (locus #1) in diverse normal and tumor specimens.

Supplementary Figure 4. ETS1 inhibits the proliferation and growth of MCF-7 breast cancer cells *in vitro* as well as *in vivo*.

Supplementary Figure 5. Bioinformatic analysis of promoter regions of representative ETS1 target genes

Supplementary Figure 6. Full Immuno-blots of the experiments

Supplementary Table 1. Primer sequences used for qRT-PCR

Supplementary Table 2. Primer sequences used for ChIP-PCR.

Supplementary Table 3. Correlation between methylation value of CpG site and *ETS1* expression in normal and tumor specimens.

Supplementary Table 4. Differential expressed genes (DEGs) in RNA-seq

Supplementary Table 5. Gene Ontology of DEG

Supplementary Table 6. List of Tumor suppressor genes (TSGs)

Supplementary Table 7. Overlap DEGs with TSGs

Supplementary Figure 1.

A

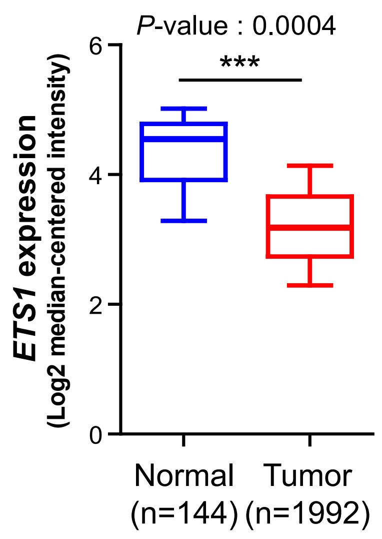

B

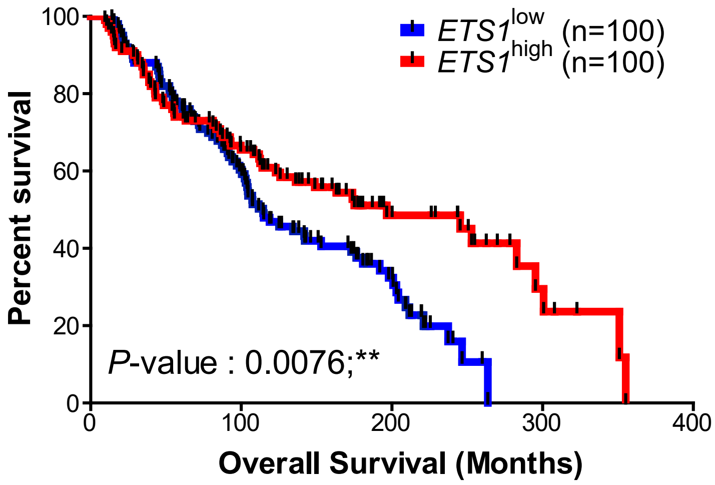

	$ETS1^{low}$	$ETS1^{high}$
# samples	100	100
# censored subjects	30	47
# deaths/events	70	53
Median survival	114.533	196.567

C

	Triple negative	Non-triple negative	Total
Total number	299	1605	1904
Death number	161	942	1103
Mutation number	25	67	92
Death in Mutation	14	39	53
Mutation & Death ratio (%)	56.0	58.2	57.6
Mutation ratio (%)	8.4	4.2	4.8

D

	Triple negative	Non-triple negative	Total
Total number	116	803	919
Death number	18	78	96
Mutation number	11	68	79
Death in Mutation	1	9	10
Mutation & Death ratio (%)	9.1	13.2	12.7
Mutation ratio (%)	9.5	8.5	8.6

Supplementary Figure 1. Comparison of *ETS1* expression between normal and tumor patient specimens in publically available dataset (Curtis). (**A**) Box plot of *ETS1* gene expression between normal and breast cancer tissues in publically available Curtis data set; normal (n=144) and tumor (n=1992). ***P<0.001 (Unpaired t test). (**B**) Correlation between *ETS1* expression and survival rate. A total of 1992 samples were divided into $ETS1^{high}$ group (n = 100) and $ETS1^{low}$ (n = 100) according to *ETS1* expression levels ($ETS1^{low}$ or $ETS1^{high}$ selection criteria are each less than the lower or higher 10% of the total patient). Overall survival analysis based on *ETS1* levels in BRCA patient. The blue or red lines indicate patients with $ETS1^{low}$ (n=100) or $ETS1^{high}$ expression (n=100), respectively. *P*-value was calculated with the Log-rank test (*P*-value = 0.0076;**). (**C-D**) Correlation between genetic alteration and survival rate of *ETS1* according to breast cancer subtype in (**C**) Curtis data set and (**D**) TCGA data set.

Supplementary Figure 2.

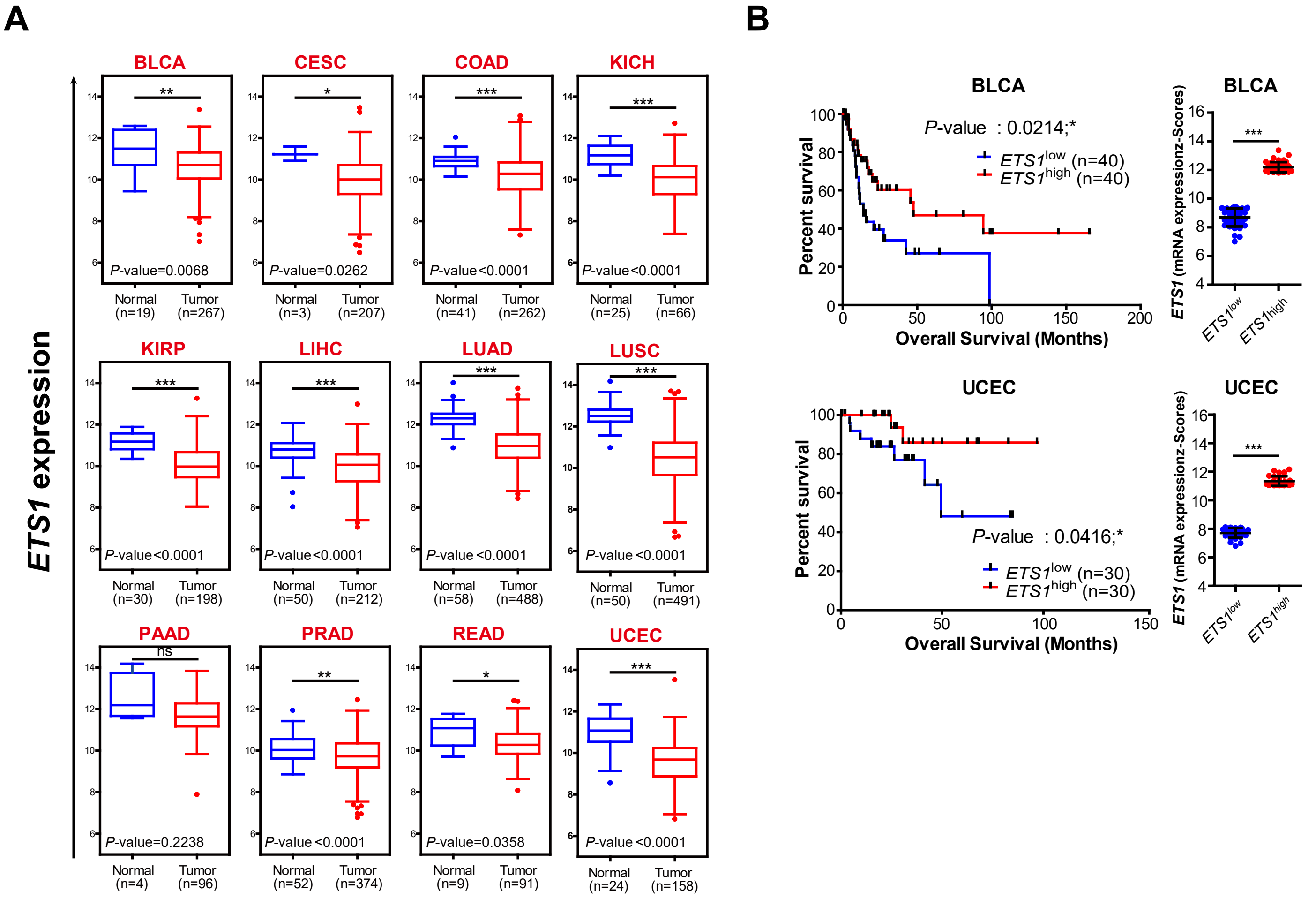

Supplementary Figure 2. Comparison of *ETS1* expression between normal and tumor patient specimens in diverse cancer types. (**A**) Tumor types that show a reduced *ETS1* expression. BLCA: Bladder Urothelial Carcinoma; CESC: Cervical squamous cell carcinoma and endocervical adenocarcinoma; COAD: colon adenocarcinoma; KICH: Kidney Chromophobe; KIRP: Kidney renal papillary cell carcinoma; LIHC: Liver hepatocellular carcinoma; LUAD: Lung adenocarcinoma; LUSC: Lung squamous cell carcinoma; PAAD: Pancreatic adenocarcinoma; PRAD: Prostate adenocarcinoma; READ: Rectum adenocarcinoma; UCEC: Uterine corpus endometrial carcinoma. *P<0.05, **P<0.01, ***P<0.001 (Unpaired t test). (**B**) Overall survival analysis based on *ETS1* levels in BLCA, UCEC. The blue or red lines indicate patients with *ETS1*^{low} or *ETS1*^{high} expression, respectively. P-value was calculated with the Log-rank test (*P<0.05) and ***P<0.001 (Unpaired t test).

Supplementary Figure 3.

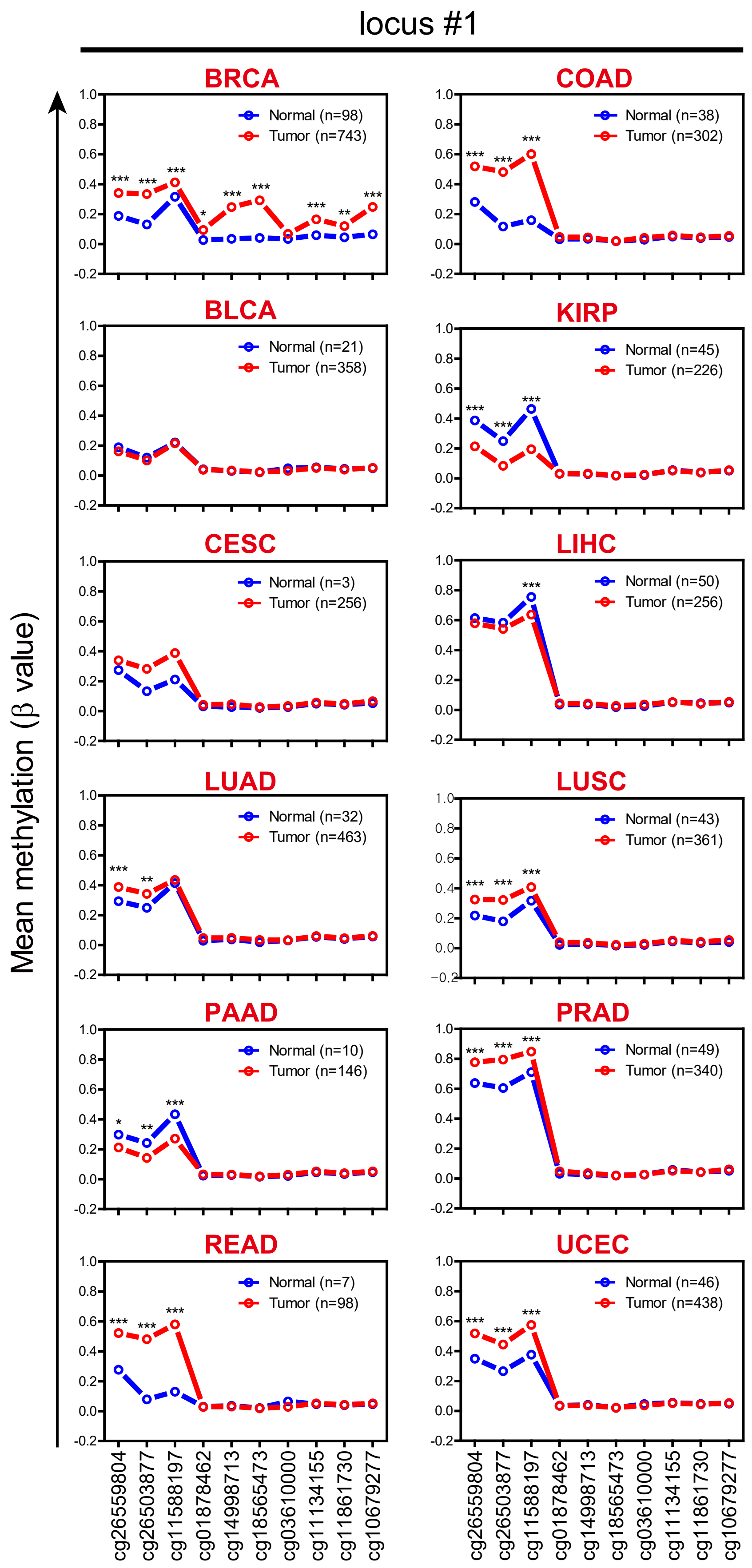

Supplementary Figure 3. DNA methylation status on ETS1 promoter (locus #1) in diverse normal and tumor specimens. Analysis of mean methylation β-value on 10 CpG sites of *ETS1* locus #1 between normal (n=98) and BRCA patient (n=743) specimens by TCGA Wanderer tool. The blue or red line represents normal or BRCA specimen, respectively. *P<0.05, **P<0.01, ***P<0.001 (two-way ANOVA with Bonferroni test).

Supplementary Figure 4.

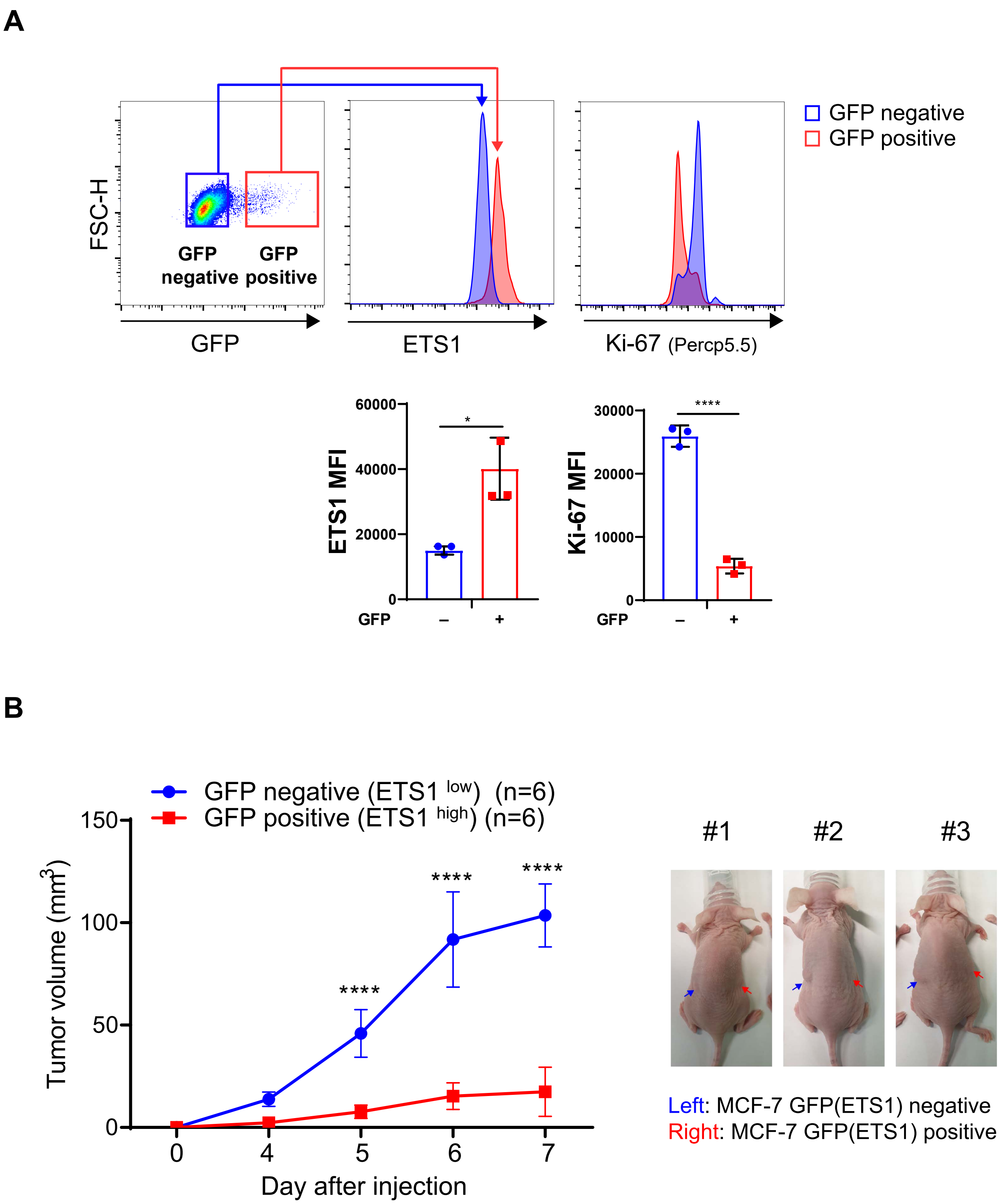

Supplementary Figure 4. ETS1 inhibits the proliferation and growth of MCF-7 breast cancer cells *in vitro* as well as *in vivo*. (**A**) MCF-7 cells (ER+, PR+ and HER2-; no ETS1 expression) were transfected with Human cDNA ETS1 GFP vector, and transfected cells were sorted according to GFP -expression. Representative data from three independent experiments. Flow cytometry analysis for ETS1 protein levels and cell proliferation (Ki -67) in GFP negative and positive. *P<0.05, **P<0.01, ***P<0.001 ****P<0.0001 (Unpaired t test). (**B**) Tumor growth curves in nude mice after injection of GFP negative (left) and positive (right) MCF-7 cells. Tumor volumes were measured. Images of representative tumor-bearing mice. Blue arrow: MCF-7 GFP(ETS1) negative; Red arrow: MCF-7 GFP(ETS1) positive. *P<0.05, **P<0.01, ***P<0.001 ****P<0.0001 (two-way ANOVA with Bonferroni test). Data is representative of three independent experiments.

Supplementary Figure 5.

A

ADAMTS9

> hg19 chr3:64673012-64674054 1243bps

TAAAATAAAACATGACGCTATCTGGTGCCCCCAATTACTAAGTTACTTTAGTTTGCGGCGTGGG
AGTGGGGATGGGGATATATCTGGCAACAGCAAGGTAGGGGGGGGTTGCCTAAATTCGTTTCC
ATAGTGTCCCTCCCTAGCAATTGGTTGGGGATGACCCAAAGAAGGGAGAGGCTGCAAAGCGG
GAGATAATTCTTTCTAGGAAAAGGAGAGAAGCCTCCGCTGCGGGGTGCCCCTGCCCAGGAGC
GAGGACCGGGAGGCGGCGTCGGGGCCGGCGGGGTCCCGGGGGCCGGAGCCTGGTTACCTT
GCCTCGGGTGCAGCCTGTCCTTGCGCACGGCCGCCGCGGCGTCTGGGCTCCCCATCTCGGC
CAGGTCCCGCACCAGGAGCGTTAGCAG**TGTGGCCCAGGATACAAACTG**CATGGTGCTTCCCA
CCCCTCCCTCCGCTGCCCCCACCCCCCTCCCTCCTGCCCTCCTTGGCTGCGGCGGCGACGC
GAGGCAGCGGCCGTGGAGAGCGCGCGGAGCCCGGCGCCCGCCGCCAACTTTT**GACTTTAGG**
AGTCGCTGAGGTCTCGCTGCGAGGGTCCCGTCTGCGCTCGGCTGAGCAACGCCGCCGCCTG
CCGAGAGCTGAGCCGCTCGGGCCGCAGGAGGAGCCGGAGGAGCAGGAGGAGGAGGAGGAC
TGGGGCTCGGCTGCTTGGCCGCATAATGCCCAGCGAGCGGGCAGGAGAAGGCGAGGAACTT
GCGCTCCGAGGCGCGCCGGGCGCCCTGTGCTGGCCGGGATAGCTGAGCGGCTTCTTGAATG
GGGGGCTGGGGGGGCGGAGGCGGGGGGGCCGCGGGTCCACAGCCTCTCAAATGCCCCCGG
TGCACGCCTCTAAGAGGAGGAGAGGGGAGGAGAAAGCGAGACGAACGGGGACCGCCTCCTT
CCAGACCATGTCCCCTCCTCGGCCGGCCCGTGCGGGACTCGCAGCCGGAGGCCCTGCCGGC
TGCAAGAGGCGGAGGCCAGAGGCGTCACCAGCGCCGGGGCAGCTGTTCCTGGTCCCCGCCA
CCTCGCTGAAGTGGGGTTTTCTGAGATTCCCTGCCCCGGGGAGGGAAAGCAGGGAAAGTTCT
CTCCCTTTCCTGGCGCGCGTGCAGCCACTTCCCCTCTCGTCTTTTTCCTCTCCTCTCCTCTCTC
TCCTCCCTTCCGAGTCTCTTTTTCCTCCTCCCCCTTGTCTCTCTGCTGCTGTGCCTCTCGGACT
CTGTCCCTTCTC

Red: ETS1 binding sites
Green: ChIP primer

B

NOTCH1

> hg19 chr9:139440638-139440858 421bps

CCCGCCCCGCCCGGCCCCACCCCCGCCGGTCCCCCCGCCCCCTCCGCGGCCACCTCCTCC
CGCACGCCCCGTCCGCCTTTGCGCCTCCCCGAGCTGAGCCGCGCGTCCCGAGCCCAGCCT
CTCCGGGGAGGAGGGACCCGGCGGCGCGGCGTGGGTCGGGAGCGGGC**GCCTGGGACTAC**
TTCTCGTTTGAAAGTTTTCAGAGGCCAAAAGTTTGAGCCGGGGCTGCGGAAGGATCCGGCTC
TGGCGGTTGCCGCGCTCGGCGACAAC**TGGCGACTGAAATTTGCATG**TGAATAAGCGGAGGA
GCCCGGGGCGGAATGGGGAGGGCGAGGCCGCGGACAGCCGTGTGCACCGTGCGCTGGGC
CGGGCGGGGAGCAGCTGAGGCCACGTTGGGGGACCCCGGGCCCGGGGCAGGGCTCTGGG
A

C

TXNIP

> hg19 chr1:145438265-145438510 446bps

GGCTAGGTTTTAGGGTCAGTGGGATCCTCCTTCCACTGGACCCGGGAGAAGACGCTCAACAG
CCCCCTCCTTCCCCTCCTTCCTCTCCTTCCTCTCCTTCCCCCCTCCCTGCGCCGCTCCAGAG
CGCAACAACCATTTTCCCAGCCAGGAGCACACCGTGTCCACGCGCCAC**AGCGATCTCACTGA**
TTGGTCGGGCTCCTGGTAAACAAGGACCGGGCAGCCAATGGGAGGGATGTGCACGAGGGCA
GCACGAGCCTCCGGGCCAGCGCTCGCGTGGCTCTTCTGGCCCGGGCTACTATATAGAGACG
TTTCCGCCTCCTGCTTGAAACTAACCCCTCTTTTTCTCCAAAGGAGTGCTTGTGGAGATCGGA
TCTTTTCTCCAGCAATTGGGGGAAAGAAGGCTTTTTCTCTGACTTCGCTTAGTGTAACCAGCG
GCGTATATTTTT

D

STAT5A

> hg19 chr17:40437576-40437785 410bps

ATTTGGGGGTGAAGAGCAGTTAGGGGCAAGGAGGCCAGCCTCGGACCGCAGCTCAGTCAGAC
CCTTGACAGTTTCTGATCTTCCTGAACCCCCATCCCGTGGCCTCAGTTTTCTCCTCCACAGCCT
GGTTCCCCAAAATCACCAGGGCA**CTTTGTGGTGGGAAATCACCC**ACACAAGGGGACAAACAAG
GGGACTGAAAGCAAAAAGAAACCCCAAATTTGACATTTCTGGGTAGTTGTGTTATCTGGAGCCA
CGACTACAGTCACCTCTTCCCAACCCAACTCTGACACCCTTCCCTCTACTCCCTTGGGTGCCCT
TTGCCCATCCTGGGAAAGTTCCATAGCTCCTCCTCCTCCCCACTGCATCTCAAAGGGAGAACAG
GCCTGAAGAAATGGAGAGAAACCCAGACA

Supplementary Figure 5. Bioinformatic analysis of promoter regions of representative ETS1 target genes (**A**) *ADAMTS9*, (**B**) *NOTCH1*, (**C**) *TXNIP* and (**D**) *STAT5A* promoter sequences were analyzed by ECR browser

Supplementary Figure 6.

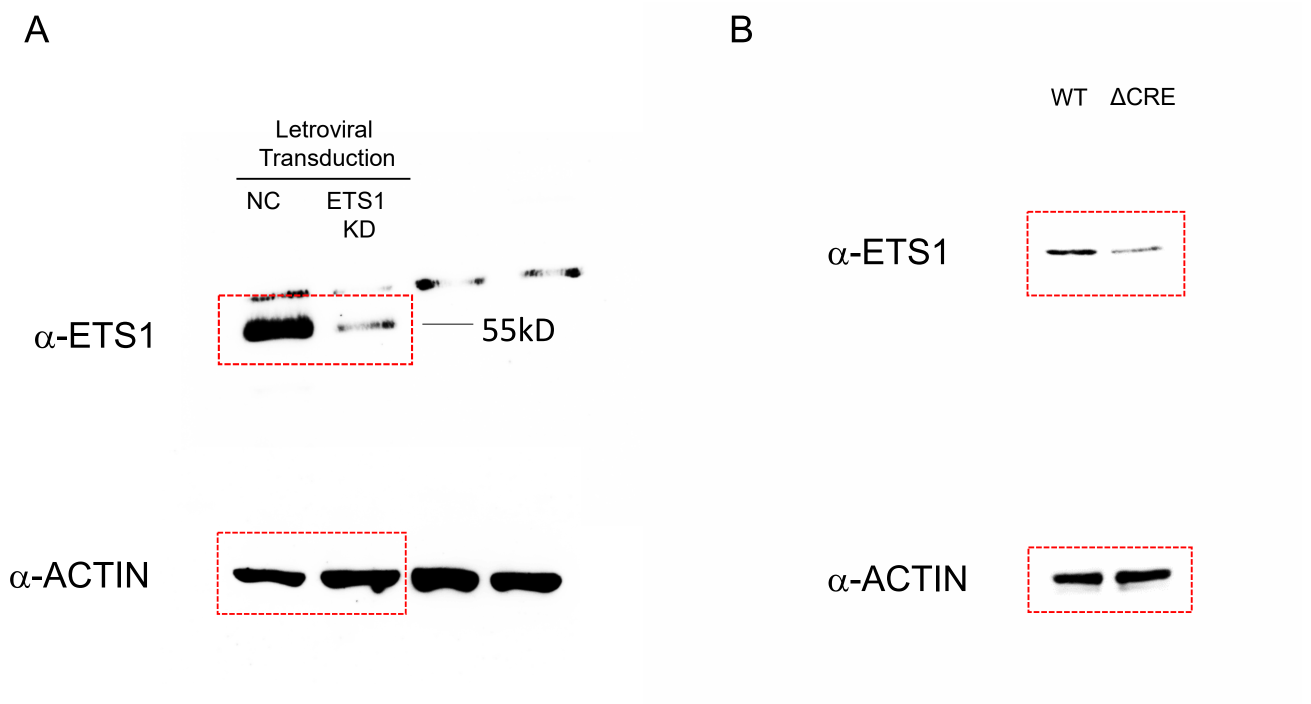

Supplementary Figure 6. Full Immuno-blots of the experiments shown in (**A**) Figure 3A (**B**) Figure 3C. Uncropped images are labeled as in the main text and red dot square indicate protein bands of interest.
